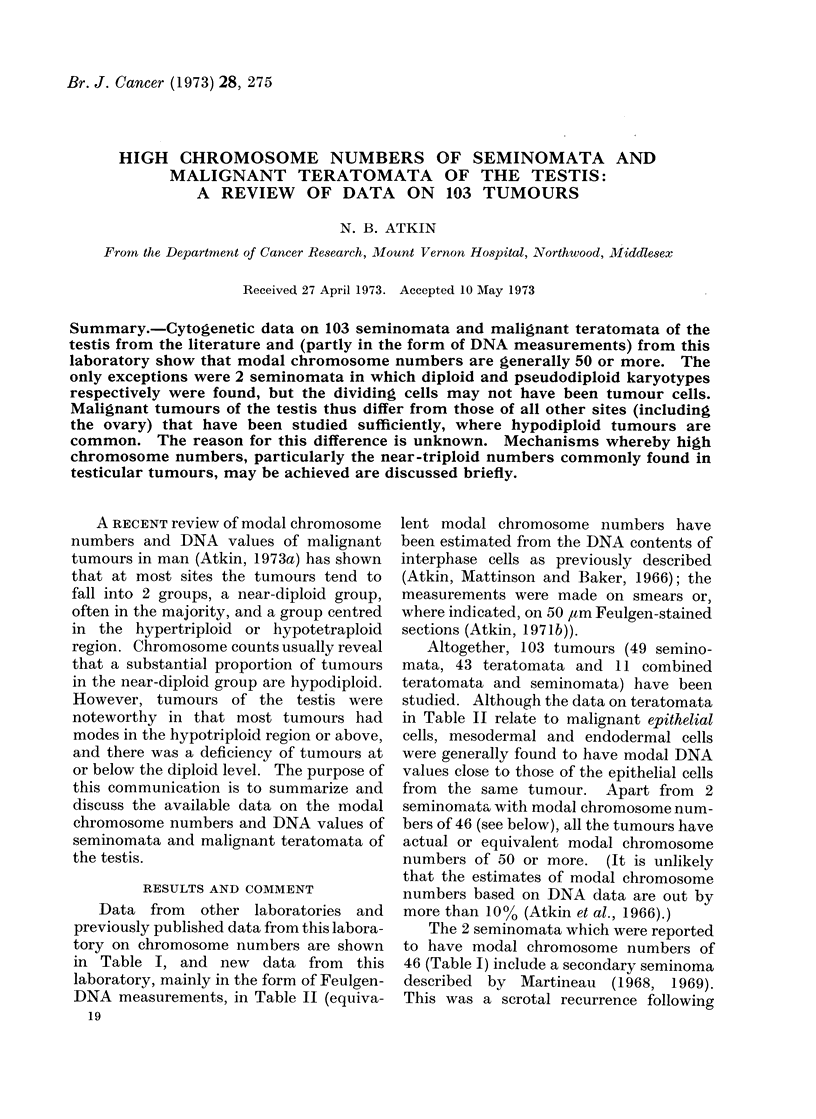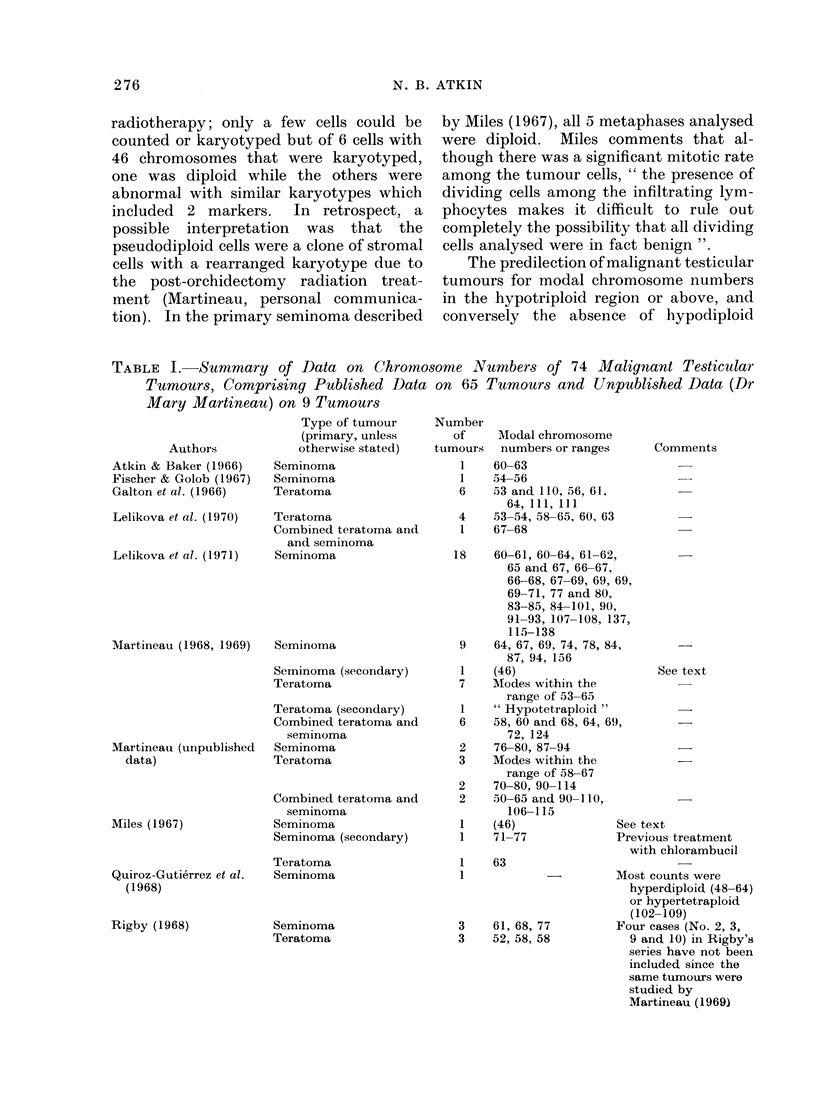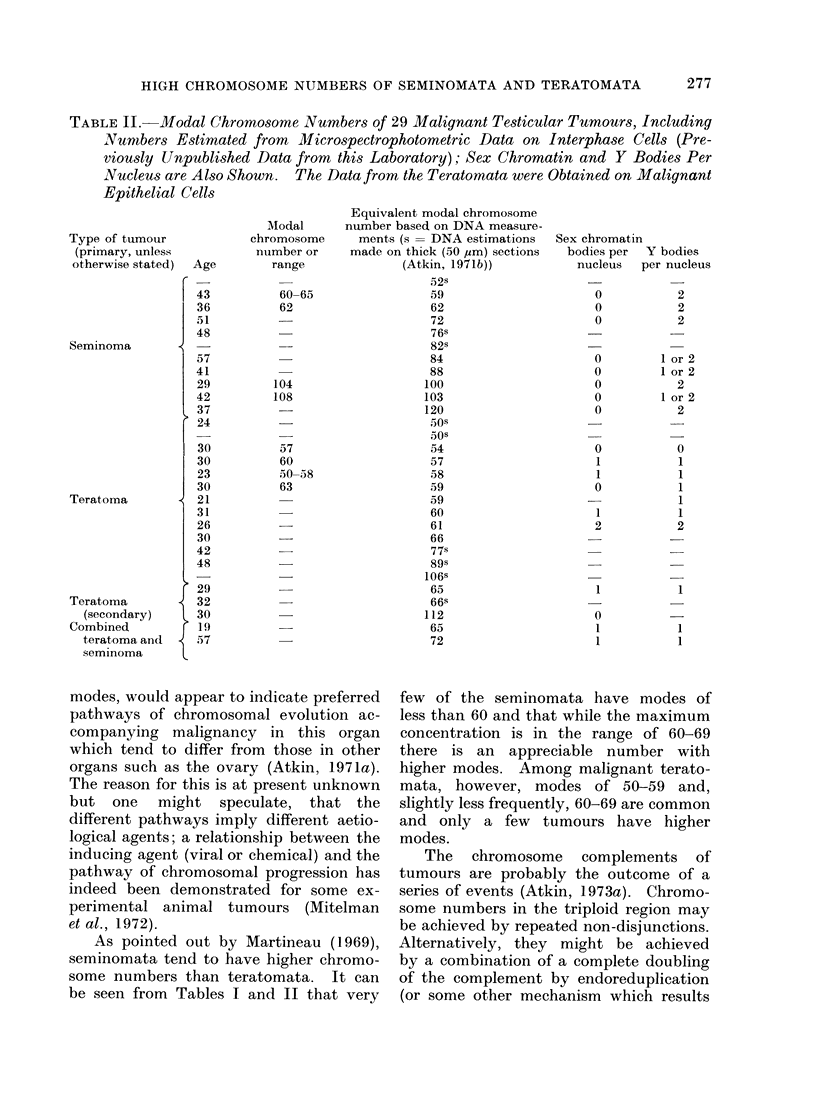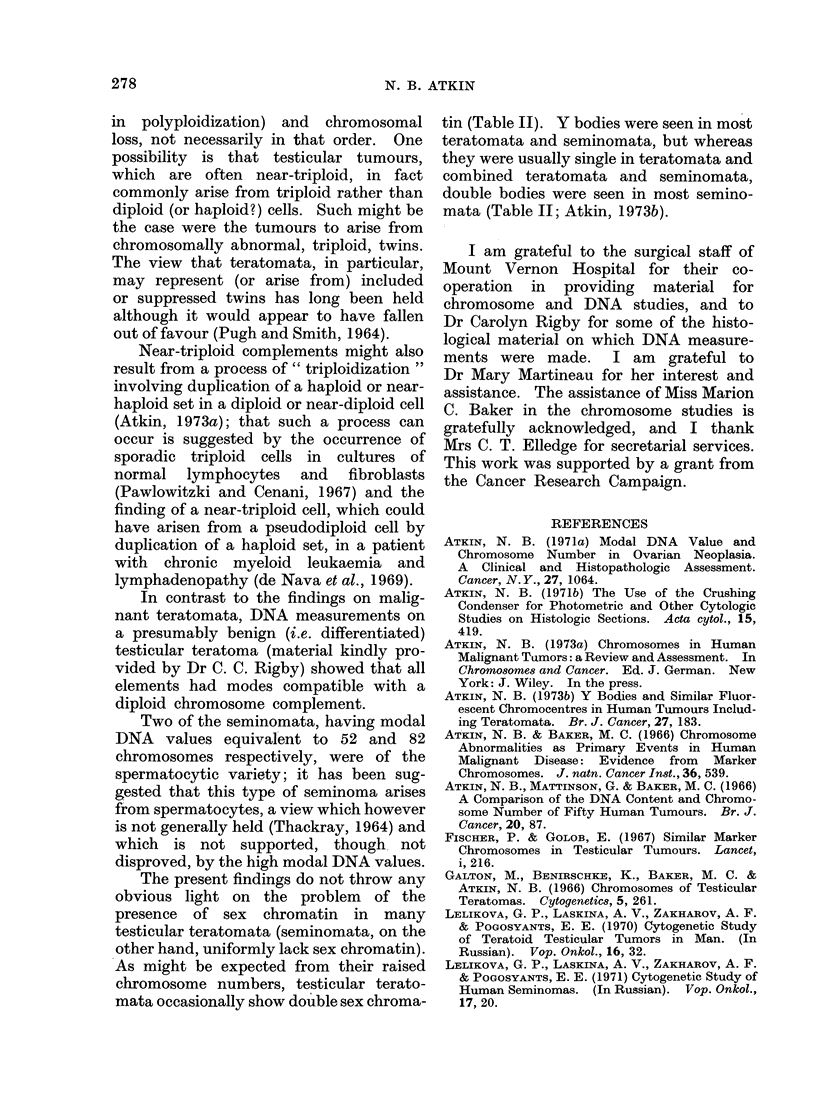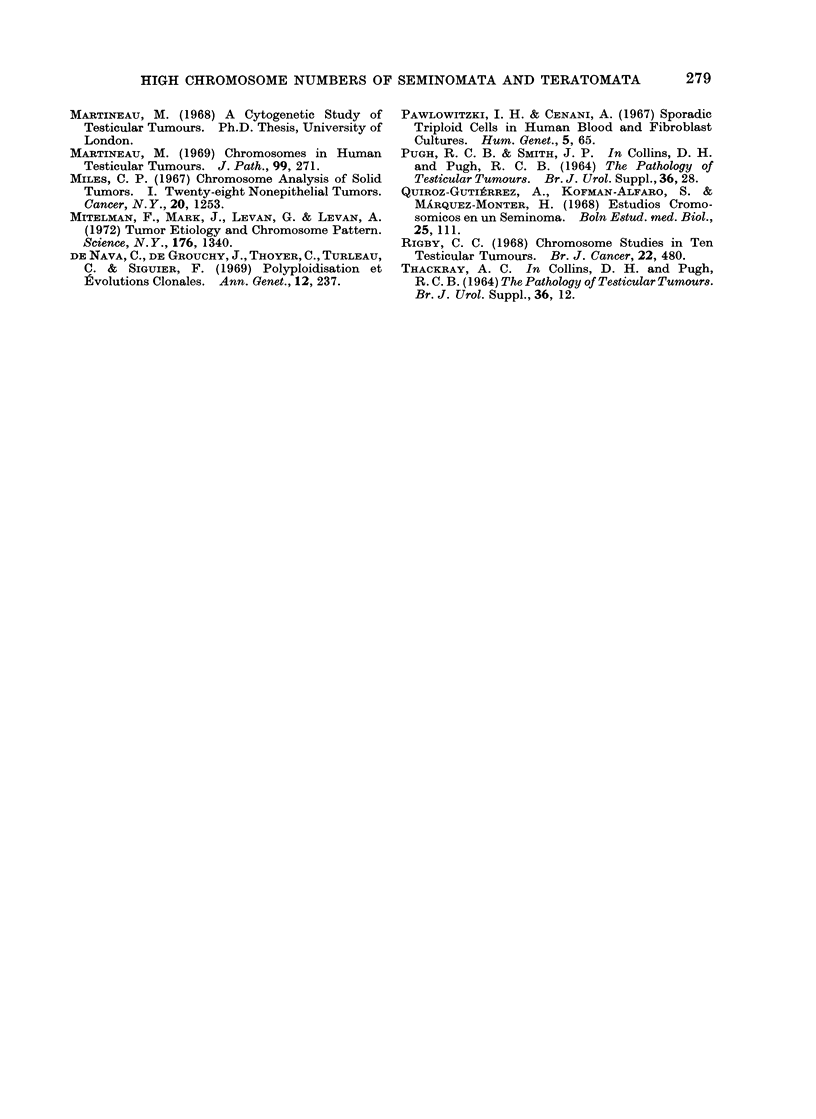# High Chromosome Numbers of Seminomata and Malignant Teratomata of the Testis: A Review of Data on 103 Tumours

**DOI:** 10.1038/bjc.1973.148

**Published:** 1973-09

**Authors:** N. B. Atkin

## Abstract

Cytogenetic data on 103 seminomata and malignant teratomata of the testis from the literature and (partly in the form of DNA measurements) from this laboratory show that modal chromosome numbers are generally 50 or more. The only exceptions were 2 seminomata in which diploid and pseudodiploid karyotypes respectively were found, but the dividing cells may not have been tumour cells. Malignant tumours of the testis thus differ from those of all other sites (including the ovary) that have been studied sufficiently, where hypodiploid tumours are common. The reason for this difference is unknown. Mechanisms whereby high chromosome numbers, particularly the near-triploid numbers commonly found in testicular tumours, may be achieved are discussed briefly.


*Br. J. Cancer* (1973) **28**, 275

### HIGH CHROMOSOME NUMBERS OF SEMINOMATA AND MALIGNANT TERATOMATA OF THE TESTIS: A REVIEW OF DATA ON 103 TUMOURS

N. B. ATKIN

*From the Department of Cancer Research, Mount Vernon Hospital, Northwood, Middlesex*

Received 27 April 1973. Accepted 10 May 1973

**Summary.—Cytogenetic data on 103 seminomata and malignant teratomata of the testis from the literature and (partly in the form of DNA measurements) from this laboratory show that modal chromosome numbers are generally 50 or more. The only exceptions were 2 seminomata in which diploid and pseudodiploid karyotypes respectively were found, but the dividing cells may not have been tumour cells. Malignant tumours of the testis thus differ from those of all other sites (including the ovary) that have been studied sufficiently, where hypodiploid tumours are common. The reason for this difference is unknown. Mechanisms whereby high chromosome numbers, particularly the near-triploid numbers commonly found in testicular tumours, may be achieved are discussed briefly.**

A RECENT review of modal chromosome numbers and DNA values of malignant tumours in man (Atkin, 1973*a*) has shown that at most sites the tumours tend to fall into 2 groups, a near-diploid group, often in the majority, and a group centred in the hypertriploid or hypotetraploid region. Chromosome counts usually reveal that a substantial proportion of tumours in the near-diploid group are hypodiploid. However, tumours of the testis were noteworthy in that most tumours had modes in the hypotriploid region or above, and there was a deficiency of tumours at or below the diploid level. The purpose of this communication is to summarize and discuss the available data on the modal chromosome numbers and DNA values of seminomata and malignant teratomata of the testis.

### RESULTS AND COMMENT

Data from other laboratories and previously published data from this laboratory on chromosome numbers are shown in Table I, and new data from this laboratory, mainly in the form of Feulgen-DNA measurements, in Table II (equivalent modal chromosome numbers have been estimated from the DNA contents of interphase cells as previously described (Atkin, Mattinson and Baker, 1966); the measurements were made on smears or, where indicated, on 50 μm Feulgen-stained sections (Atkin, 1971*b*)).

Altogether, 103 tumours (49 seminomata, 43 teratomata and 11 combined teratomata and seminomata) have been studied. Although the data on teratomata in Table II relate to malignant *epithelial* cells, mesodermal and endodermal cells were generally found to have modal DNA values close to those of the epithelial cells from the same tumour. Apart from 2 seminomata with modal chromosome numbers of 46 (see below), all the tumours have actual or equivalent modal chromosome numbers of 50 or more. (It is unlikely that the estimates of modal chromosome numbers based on DNA data are out by more than 10% (Atkin *et al.*, 1966).)

The 2 seminomata which were reported to have modal chromosome numbers of 46 (Table I) include a secondary seminoma described by Martineau (1968, 1969). This was a scrotal recurrence following radiotherapy; only a few cells could be counted or karyotyped but of 6 cells with 46 chromosomes that were karyotyped, one was diploid while the others were abnormal with similar karyotypes which included 2 markers. In retrospect, a possible interpretation was that the pseudodiploid cells were a clone of stromal cells with a rearranged karyotype due to the post-orchidectomy radiation treatment (Martineau, personal communication). In the primary seminoma described by Miles (1967), all 5 metaphases analysed were diploid. Miles comments that although there was a significant mitotic rate among the tumour cells, " the presence of dividing cells among the infiltrating lymphocytes makes it difficult to rule out completely the possibility that all dividing cells analysed were in fact benign ".

The predilection of malignant testicular tumours for modal chromosome numbers in the hypotriploid region or above, and conversely the absence of hypodiploid

TABLE I.—*Summary of Data on Chromosome Numbers of 74 Malignant Testicular Tumours, Comprising Published Data on 65 Tumours and Unpublished Data (Dr Mary Martineau) on 9 Tumours*

| Authors | Type of tumour (primary, unless otherwise stated) | Number of tumours | Modal chromosome numbers or ranges | Comments |
|---|---|---|---|---|
| Atkin & Baker (1966) | Seminoma | 1 | 60–63 | — |
| Fischer & Golob (1967) | Seminoma | 1 | 54–56 | — |
| Galton *et al.* (1966) | Teratoma | 6 | 53 and 110, 56, 61, 64, 111, 111 | — |
| Lelikova *et al.* (1970) | Teratoma | 4 | 53–54, 58–65, 60, 63 | — |
| | Combined teratoma and and seminoma | 1 | 67–68 | — |
| Lelikova *et al.* (1971) | Seminoma | 18 | 60–61, 60–64, 61–62, 65 and 67, 66–67, 66–68, 67–69, 69, 69, 69–71, 77 and 80, 83–85, 84–101, 90, 91–93, 107–108, 137, 115–138 | — |
| Martineau (1968, 1969) | Seminoma | 9 | 64, 67, 69, 74, 78, 84, 87, 94, 156 | — |
| | Seminoma (secondary) | 1 | (46) | See text |
| | Teratoma | 7 | Modes within the range of 53–65 | — |
| | Teratoma (secondary) | 1 | " Hypotetraploid " | — |
| | Combined teratoma and seminoma | 6 | 58, 60 and 68, 64, 69, 72, 124 | — |
| Martineau (unpublished data) | Seminoma | 2 | 76–80, 87–94 | — |
| | Teratoma | 3 | Modes within the range of 58–67 | — |
| | | 2 | 70–80, 90–114 | |
| | Combined teratoma and seminoma | 2 | 50–65 and 90–110, 106–115 | — |
| Miles (1967) | Seminoma | 1 | (46) | See text |
| | Seminoma (secondary) | 1 | 71–77 | Previous treatment with chlorambucil |
| | Teratoma | 1 | 63 | — |
| Quiroz-Gutiérrez *et al.* (1968) | Seminoma | 1 | — | Most counts were hyperdiploid (48–64) or hypertetraploid (102–109) |
| Rigby (1968) | Seminoma | 3 | 61, 68, 77 | Four cases (No. 2, 3, 9 and 10) in Rigby's series have not been included since the same tumours were studied by Martineau (1969) |
| | Teratoma | 3 | 52, 58, 58 | |

TABLE II.—*Modal Chromosome Numbers of 29 Malignant Testicular Tumours, Including Numbers Estimated from Microspectrophotometric Data on Interphase Cells (Previously Unpublished Data from this Laboratory); Sex Chromatin and Y Bodies Per Nucleus are Also Shown. The Data from the Teratomata were Obtained on Malignant Epithelial Cells*

| Type of tumour (primary, unless otherwise stated) | Age | Modal chromosome number or range | Equivalent modal chromosome number based on DNA measurements (s = DNA estimations made on thick (50 μm) sections (Atkin, 1971*b*)) | Sex chromatin bodies per nucleus | Y bodies per nucleus |
|---|---|---|---|---|---|
| Seminoma | — | — | 52s | — | — |
| | 43 | 60–65 | 59 | 0 | 2 |
| | 36 | 62 | 62 | 0 | 2 |
| | 51 | — | 72 | 0 | 2 |
| | 48 | — | 76s | — | — |
| | — | — | 82s | — | — |
| | 57 | — | 84 | 0 | 1 or 2 |
| | 41 | — | 88 | 0 | 1 or 2 |
| | 29 | 104 | 100 | 0 | 2 |
| | 42 | 108 | 103 | 0 | 1 or 2 |
| | 37 | — | 120 | 0 | 2 |
| Teratoma | 24 | — | 50s | — | — |
| | — | — | 50s | — | — |
| | 30 | 57 | 54 | 0 | 0 |
| | 30 | 60 | 57 | 1 | 1 |
| | 23 | 50–58 | 58 | 1 | 1 |
| | 30 | 63 | 59 | 0 | 1 |
| | 21 | — | 59 | — | 1 |
| | 31 | — | 60 | 1 | 1 |
| | 26 | — | 61 | 2 | 2 |
| | 30 | — | 66 | — | — |
| | 42 | — | 77s | — | — |
| | 48 | — | 89s | — | — |
| | — | — | 106s | — | — |
| Teratoma (secondary) | 29 | — | 65 | 1 | 1 |
| | 32 | — | 66s | — | — |
| | 30 | — | 112 | 0 | — |
| Combined teratoma and seminoma | 19 | — | 65 | 1 | 1 |
| | 57 | — | 72 | 1 | 1 |

modes, would appear to indicate preferred pathways of chromosomal evolution accompanying malignancy in this organ which tend to differ from those in other organs such as the ovary (Atkin, 1971*a*). The reason for this is at present unknown but one might speculate, that the different pathways imply different aetiological agents; a relationship between the inducing agent (viral or chemical) and the pathway of chromosomal progression has indeed been demonstrated for some experimental animal tumours (Mitelman *et al.*, 1972).

As pointed out by Martineau (1969), seminomata tend to have higher chromosome numbers than teratomata. It can be seen from Tables I and II that very few of the seminomata have modes of less than 60 and that while the maximum concentration is in the range of 60–69 there is an appreciable number with higher modes. Among malignant teratomata, however, modes of 50–59 and, slightly less frequently, 60–69 are common and only a few tumours have higher modes.

The chromosome complements of tumours are probably the outcome of a series of events (Atkin, 1973*a*). Chromosome numbers in the triploid region may be achieved by repeated non-disjunctions. Alternatively, they might be achieved by a combination of a complete doubling of the complement by endoreduplication (or some other mechanism which results in polyploidization) and chromosomal loss, not necessarily in that order. One possibility is that testicular tumours, which are often near-triploid, in fact commonly arise from triploid rather than diploid (or haploid?) cells. Such might be the case were the tumours to arise from chromosomally abnormal, triploid, twins. The view that teratomata, in particular, may represent (or arise from) included or suppressed twins has long been held although it would appear to have fallen out of favour (Pugh and Smith, 1964).

Near-triploid complements might also result from a process of " triploidization " involving duplication of a haploid or near-haploid set in a diploid or near-diploid cell (Atkin, 1973*a*); that such a process can occur is suggested by the occurrence of sporadic triploid cells in cultures of normal lymphocytes and fibroblasts (Pawlowitzki and Cenani, 1967) and the finding of a near-triploid cell, which could have arisen from a pseudodiploid cell by duplication of a haploid set, in a patient with chronic myeloid leukaemia and lymphadenopathy (de Nava *et al.*, 1969).

In contrast to the findings on malignant teratomata, DNA measurements on a presumably benign (*i.e.* differentiated) testicular teratoma (material kindly provided by Dr C. C. Rigby) showed that all elements had modes compatible with a diploid chromosome complement.

Two of the seminomata, having modal DNA values equivalent to 52 and 82 chromosomes respectively, were of the spermatocytic variety; it has been suggested that this type of seminoma arises from spermatocytes, a view which however is not generally held (Thackray, 1964) and which is not supported, though not disproved, by the high modal DNA values.

The present findings do not throw any obvious light on the problem of the presence of sex chromatin in many testicular teratomata (seminomata, on the other hand, uniformly lack sex chromatin). As might be expected from their raised chromosome numbers, testicular teratomata occasionally show double sex chromatin (Table II). Y bodies were seen in most teratomata and seminomata, but whereas they were usually single in teratomata and combined teratomata and seminomata, double bodies were seen in most seminomata (Table II; Atkin, 1973*b*).

I am grateful to the surgical staff of Mount Vernon Hospital for their co-operation in providing material for chromosome and DNA studies, and to Dr Carolyn Rigby for some of the histological material on which DNA measurements were made. I am grateful to Dr Mary Martineau for her interest and assistance. The assistance of Miss Marion C. Baker in the chromosome studies is gratefully acknowledged, and I thank Mrs C. T. Elledge for secretarial services. This work was supported by a grant from the Cancer Research Campaign.

### REFERENCES

ATKIN, N. B. (1971*a*) Modal DNA Value and Chromosome Number in Ovarian Neoplasia. A Clinical and Histopathologic Assessment. *Cancer, N.Y.*, **27**, 1064.

ATKIN, N. B. (1971*b*) The Use of the Crushing Condenser for Photometric and Other Cytologic Studies on Histologic Sections. *Acta cytol.*, **15**, 419.

ATKIN, N. B. (1973*a*) Chromosomes in Human Malignant Tumors: a Review and Assessment. In *Chromosomes and Cancer*. Ed. J. German. New York: J. Wiley. In the press.

ATKIN, N. B. (1973*b*) Y Bodies and Similar Fluorescent Chromocentres in Human Tumours Including Teratomata. *Br. J. Cancer*, **27**, 183.

ATKIN, N. B. & BAKER, M. C. (1966) Chromosome Abnormalities as Primary Events in Human Malignant Disease: Evidence from Marker Chromosomes. *J. natn. Cancer Inst.*, **36**, 539.

ATKIN, N. B., MATTINSON, G. & BAKER, M. C. (1966) A Comparison of the DNA Content and Chromosome Number of Fifty Human Tumours. *Br. J. Cancer*, **20**, 87.

FISCHER, P. & GOLOB, E. (1967) Similar Marker Chromosomes in Testicular Tumours. *Lancet*, i, 216.

GALTON, M., BENIRSCHKE, K., BAKER, M. C. & ATKIN, N. B. (1966) Chromosomes of Testicular Teratomas. *Cytogenetics*, **5**, 261.

LELIKOVA, G. P., LASKINA, A. V., ZAKHAROV, A. F. & POGOSYANTS, E. E. (1970) Cytogenetic Study of Teratoid Testicular Tumors in Man. (In Russian). *Vop. Onkol.*, **16**, 32.

LELIKOVA, G. P., LASKINA, A. V., ZAKHAROV, A. F. & POGOSYANTS, E. E. (1971) Cytogenetic Study of Human Seminomas. (In Russian). *Vop. Onkol.*, **17**, 20.

MARTINEAU, M. (1968) A Cytogenetic Study of Testicular Tumours. Ph.D. Thesis, University of London.

MARTINEAU, M. (1969) Chromosomes in Human Testicular Tumours. *J. Path.*, **99**, 271.

MILES, C. P. (1967) Chromosome Analysis of Solid Tumors. I. Twenty-eight Nonepithelial Tumors. *Cancer, N.Y.*, **20**, 1253.

MITELMAN, F., MARK, J., LEVAN, G. & LEVAN, A. (1972) Tumor Etiology and Chromosome Pattern. *Science, N.Y.*, **176**, 1340.

DE NAVA, C., DE GROUCHY, J., THOYER, C., TURLEAU, C. & SIGUIER, F. (1969) Polyploidisation et Évolutions Clonales. *Ann. Genet.*, **12**, 237.

PAWLOWITZKI, I. H. & CENANI, A. (1967) Sporadic Triploid Cells in Human Blood and Fibroblast Cultures. *Hum. Genet.*, **5**, 65.

PUGH, R. C. B. & SMITH, J. P. *In* Collins, D. H. and Pugh, R. C. B. (1964) *The Pathology of Testicular Tumours. Br. J. Urol.* Suppl., **36**, 28.

QUIROZ-GUTIÉRREZ, A., KOFMAN-ALFARO, S. & MÁRQUEZ-MONTER, H. (1968) Estudios Cromosomicos en un Seminoma. *Boln Estud. med. Biol.*, **25**, 111.

RIGBY, C. C. (1968) Chromosome Studies in Ten Testicular Tumours. *Br. J. Cancer*, **22**, 480.

THACKRAY, A. C. *In* Collins, D. H. and Pugh, R. C. B. (1964) *The Pathology of Testicular Tumours. Br. J. Urol.* Suppl., **36**, 12.